# Synthesis, crystal structure determination, Hirshfeld surface and crystal void analyses, inter­action energy calculations and energy frameworks of *N*-(2-chloro­phen­yl)-*N*′-propano­ylthio­urea

**DOI:** 10.1107/S205698902600602X

**Published:** 2026-06-12

**Authors:** Sharatha Kumar, Tuncer Hökelek

**Affiliations:** a Department of Chemistry Yenepoya Institute of Arts Science Commerce and Management Mangaluru Yenepoya University (Deemed to be university) 575013 Karnataka India; b Hacettepe University, Department of Physics, 06800 Beytepe-Ankara, Türkiye; University of Neuchâtel, Switzerland

**Keywords:** propino­yl, thio­urea, 2-chloro­phen­yl, crystal structure

## Abstract

The title compound, C_10_H_11_ClN_2_S, consists of a chloro­phenyl ring and a propanoyl moiety bridged over a thio­urea functional group. The dihedral angle between the planar propinoyl and thio­urea groups is 8.33 (14)°, and they are oriented at 48.50 (14) and 56.09 (6)° with respect to the phenyl ring. The intra­molecular N—H⋯O hydrogen bond forms an S(6) ring motif. In the crystal, N—H⋯S hydrogen bonds link two mol­ecules, enclosing *R*^2^_2_(8) ring motifs, into centrosymmetric dimers. π–π stacking inter­actions help to consolidate the packing. Hirshfeld surface analysis revealed that the most important contributions for crystal packing are H⋯H (39.2%), H⋯Cl/Cl⋯H (15.8%), H⋯S/S⋯H (14.2%) and H⋯C/C⋯H (9.9%) inter­actions. The volume of the crystal voids and the percentage of free space were calculated to be 105.62 Å^3^ and 9.33%, showing that there is no large cavity in the crystal packing. Computational methods indicated an N—H⋯S hydrogen-bonding energy of −53.5 kJ mol^−1^. Evaluations of the electrostatic, dispersion and total energy frameworks indicate that the crystal cohesion is dominated by electrostatic energy contributions.

## Abstract 2

The title compound, C_10_H_11_ClN_2_S, consists of a chloro­phenyl ring and a propanoyl moiety bridged over a thio­urea functional group. In the crystal, N—H⋯S hydrogen bonds link the mol­ecules into centrosymmetric dimers. π–π stacking inter­actions may help to consolidate the packing.

## 1. Chemical context

Thio­urea is an important organic scaffold widely used in the development of therapeutic and industrially relevant mol­ecules. Several applications have been reported, and researchers continue to explore thio­urea derivatives in agriculture, gold recovery, analytical chemistry, and medicine (Rizki *et al.*, 2019 ▸; Shakeel *et al.*, 2016 ▸). Thio­urea derivatives exhibit diverse biological activities, including anti­cancer (Nammalwar *et al.*, 2013 ▸), anti­thyroid, anti­malarial (Mishra & Batra, 2013 ▸), anti­fungal, anti­viral, anti­microbial, anti­oxidant, anti-allergic, anti-inflammatory, anti­septic, anti-leishmanial (Viana *et al.*, 2017 ▸), and anti-hypertensive effects.

The thio­urea pharmacophore possesses unique chemical features, such as hydrogen-bonding groups (NH), a complementary sulfur site, and auxiliary binding positions at the 1,3-substituents, which enable strong and versatile inter­actions with biological targets (Mishra & Batra, 2013 ▸). Sulfur acts as a weak hydrogen-bond acceptor, while the bidentate nature of the thio­urea protons enhances hydrogen bonding, making thio­urea derivatives highly effective in medicinal chemistry (Nammalwar *et al.*, 2013 ▸).

We became inter­ested in the properties and crystal structures of acyl­thio­ureas because of their notable biological activities, versatile metal-coordination behaviour, and ability to generate diverse supra­molecular hydrogen-bonding networks (Kumar *et al.*, 2012 ▸; Gowda *et al.*, 2012 ▸; Aly *et al.*, 2007 ▸; Saeed *et al.*, 2014 ▸, 2017 ▸). Numerous crystallographic investigations have demonstrated that acyl­thio­urea derivatives adopt diverse conformations consolidated through intra- and inter­molecular hydrogen bonding, particularly involving N—H⋯S and N—H⋯O inter­actions. These compounds also exhibit significant coordination versatility toward transition metals due to the presence of sulfur and carbonyl donor atoms. Detailed structural analyses of substituted acyl­thio­ureas have been reported in the literature (Arslan *et al.*, 2003 ▸; Ghosh *et al.*, 2010 ▸), highlighting the influence of the mol­ecular geometry and supra­molecular assembly on their physicochemical and biological properties.
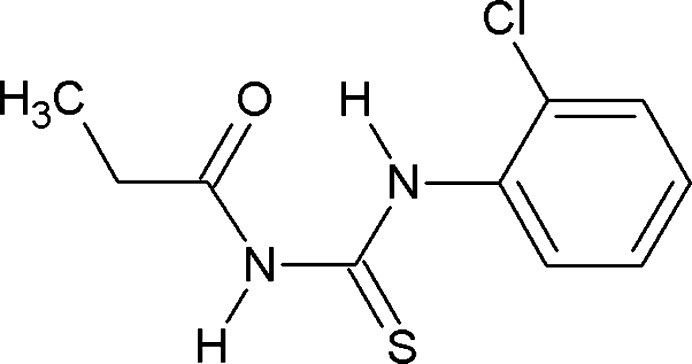


Herein, we report the mol­ecular and crystal structures of *N*-(2-chloro­phen­yl)-*N*′-propano­ylthio­urea and Hirshfeld surface (HS) and crystal void analyses and inter­action energy calculations and energy frameworks.

## 2. Structural commentary

**Figure 1:**
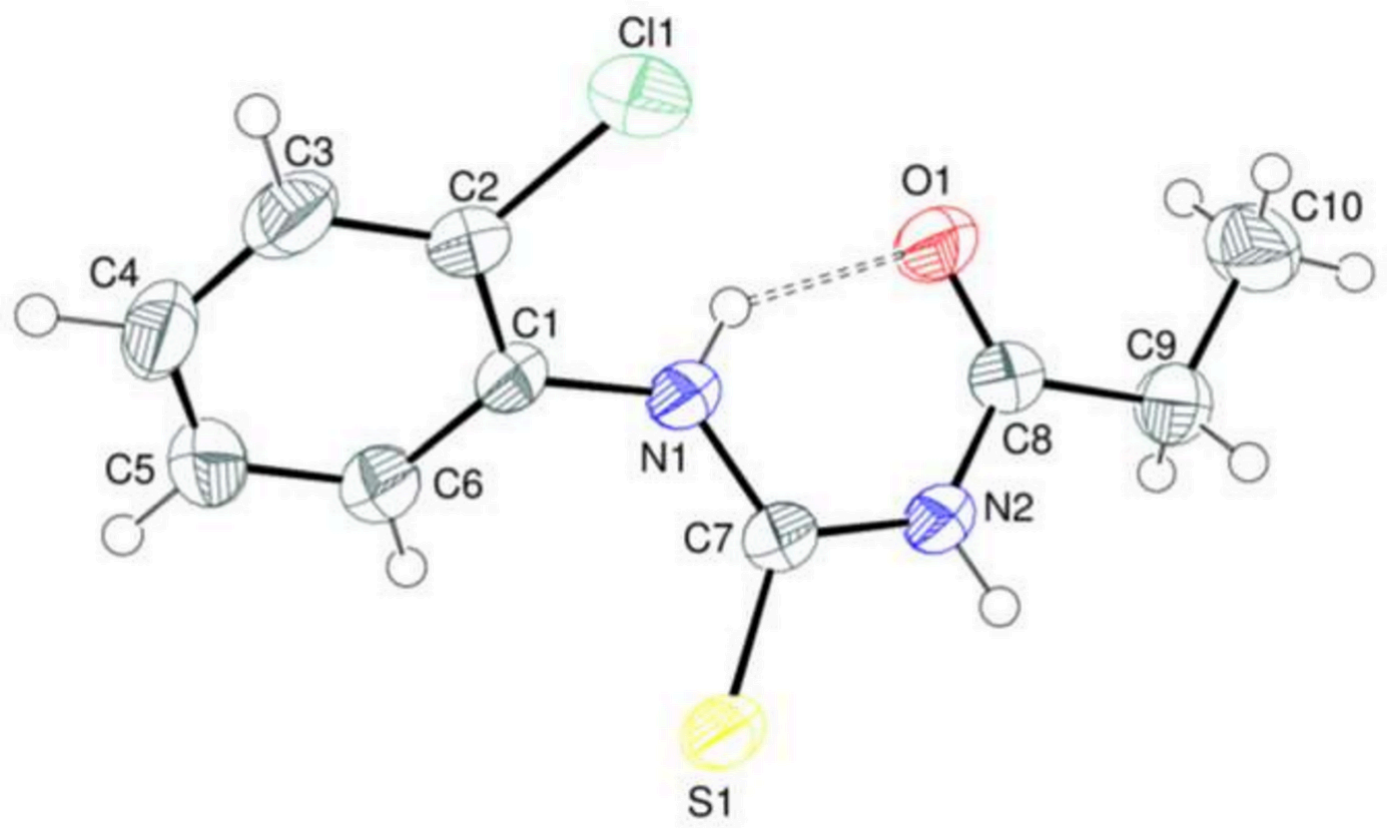
The asymmetric unit with atom-numbering scheme and 50% probability ellipsoids.

**Figure 2:**
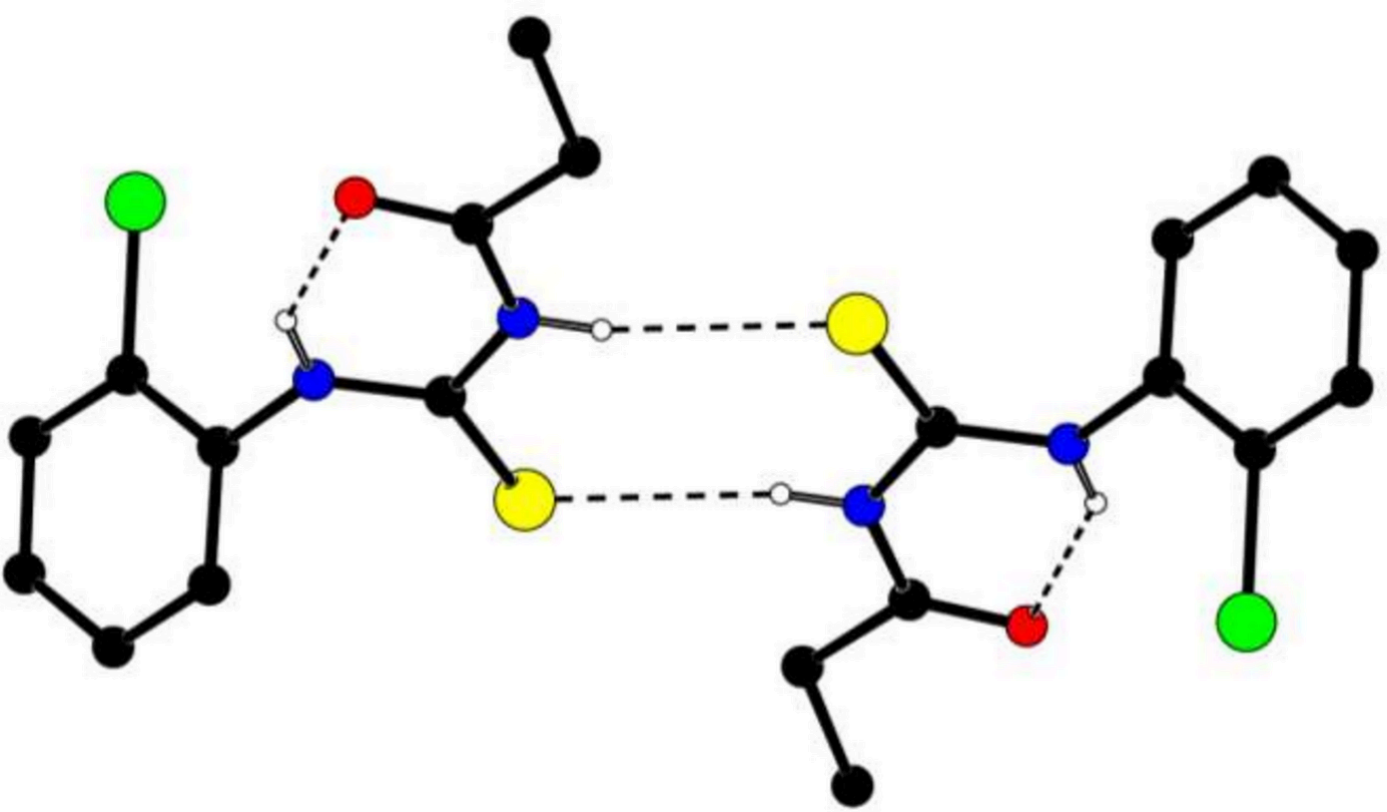
A partial packing diagram showing the intra­molecular N—H⋯O and inter­molecular N—H⋯S hydrogen bonds as dashed lines illustrating the intra­molecular *S*(6) and inter­molecular 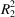
(8) ring motifs.

The title compound consists of a chloro­phenyl ring and propanoyl moiety bridged by a thio­urea functional group (Fig. 1 ▸). The planar propinoyl (O1/C8–C10) and thio­urea (S1/C7/N1/N2) groups (r.m.s. deviations of 0.039 and 0.013 Å, respectively) subtend a dihedral angle of 8.33 (14)°. The dihedral angles between the phenyl (C1–C6) ring and the propinoyl and thio­urea groups are 48.50 (14) and 56.09 (6)°, respectively. The Cl1 and N1 atoms are 0.0037 (8) and −0.0345 (23) Å away from the best plane of the phenyl ring, so they are coplanar. The bond lengths are in normal ranges (Allen *et al.*, 1987 ▸) and comparable to those in the similar compounds *N*-(3-chloro­propion­yl)-*N*′-phenyl­thio­urea (Oth­man *et al.*, 2010 ▸) and *N*-(2,6-di­methyl­phen­yl)-*N*′ propano­ylthio­urea (Yusof *et al.*, 2012 ▸). The C1—N1—C7 [126.15 (19)°] and S1—C7—N1 [125.21 (18)°] bond angles are significantly wider, while the N1—C7—N2 [115.49 (19)°] and C2—C1—C6 [118.6 (2)°] are narrowed with respect to those found in these analogous structures. An intra­molecular N—H⋯O hydrogen bond (Table 1 ▸) forms an *S*(6) ring motif (Etter *et al.*, 1990 ▸) (Fig. 2 ▸).

## 3. Supra­molecular features

In the crystal, N—H⋯S hydrogen bonds (Table 1 ▸) link the mol­ecules, enclosing 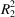
(8) ring motifs (Etter *et al.*, 1990 ▸), into centrosymmetric dimers (Fig. 2 ▸). Weak π–π stacking inter­actions between parallel phenyl rings, with a centroid-to-centroid distance of 4.1188 (17) Å, help to consolidate the packing.

**Figure 3:**
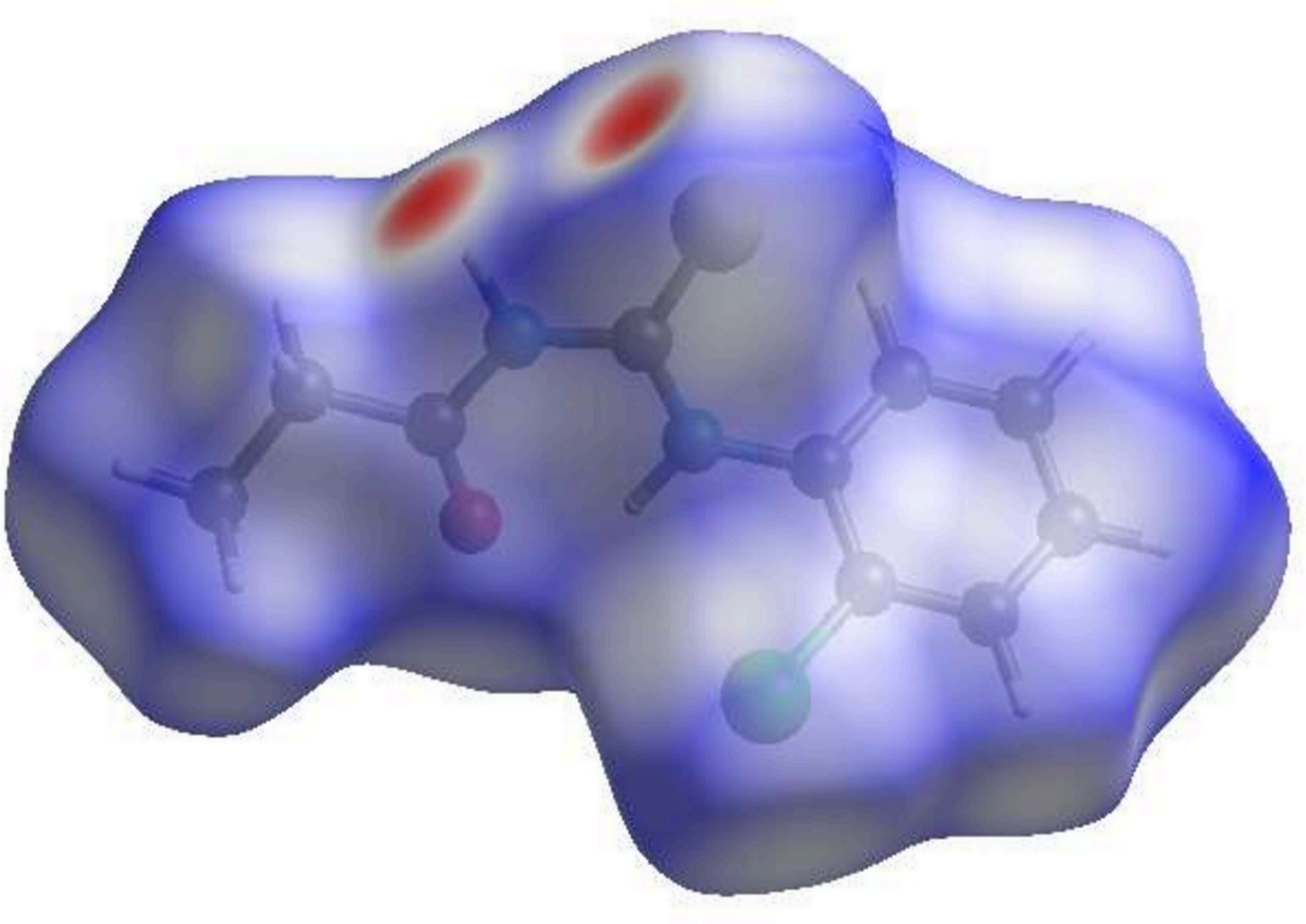
View of the three-dimensional Hirshfeld surface plotted over *d*_norm_ in the range −0.3374 to 1.1449 a.u.

**Figure 4:**
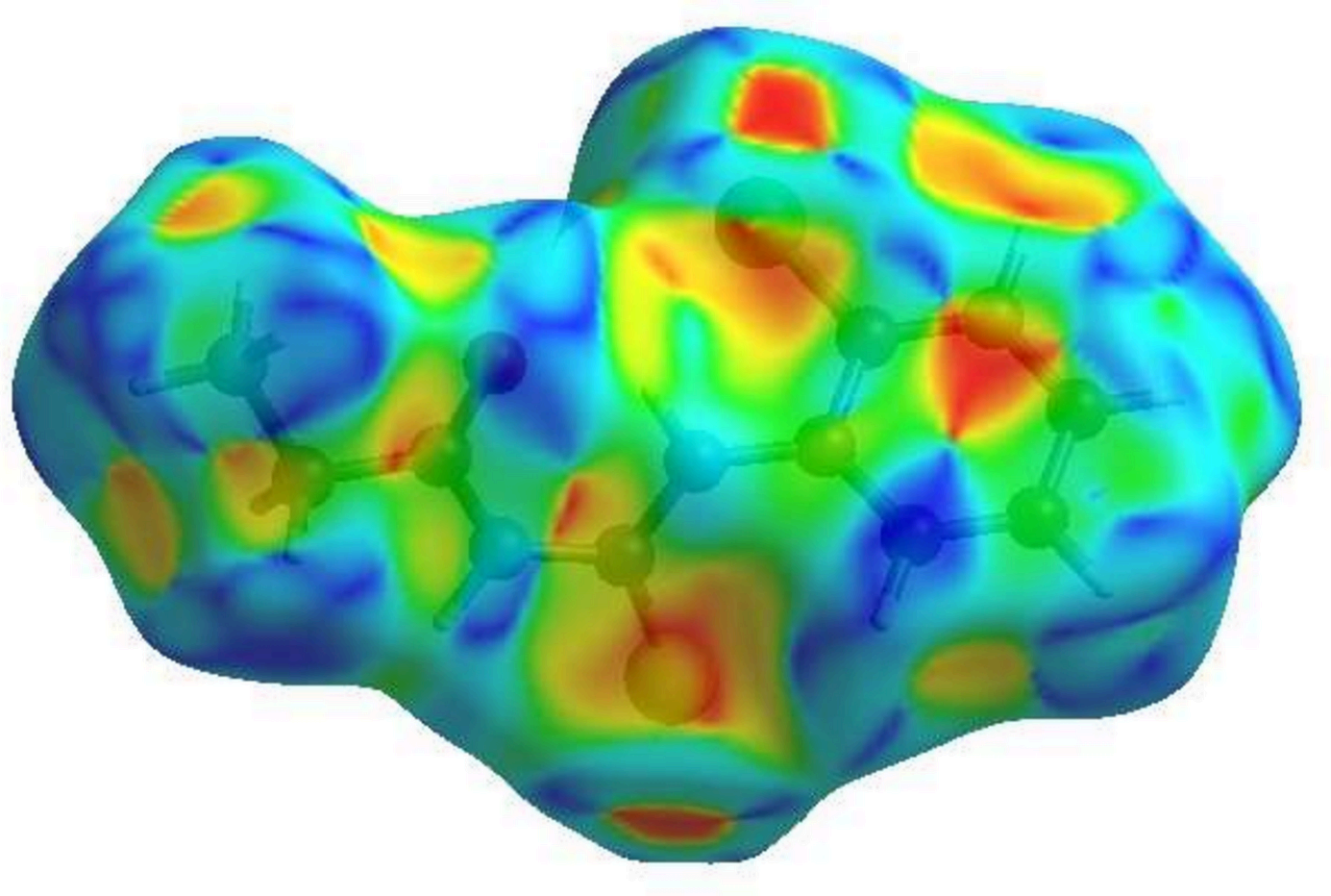
Hirshfeld surface plotted over shape-index showing the π–π inter­actions.

The inter­molecular inter­actions in the crystal were visualized by carrying out the Hirshfeld surface (HS) analysis using *CrystalExplorer* 17.5 (Spackman *et al.*, 2021 ▸). Fig. 3 ▸ shows the Hirshfeld surface with several neighboring mol­ecules in the crystal. The white surface indicates contacts with distances equal to the sum of van der Waals radii, and the red and blue colours indicate distances shorter (in close contact) or longer (distinct contacts) than the van der Waals radii, respectively. The red spots indicate their roles as the respective donors and/or acceptors atoms in hydrogen bonding, as discussed above. The π–π stacking inter­actions are shown in Fig. 4 ▸ by the presence of the adjacent red and blue triangles.

**Figure 5:**
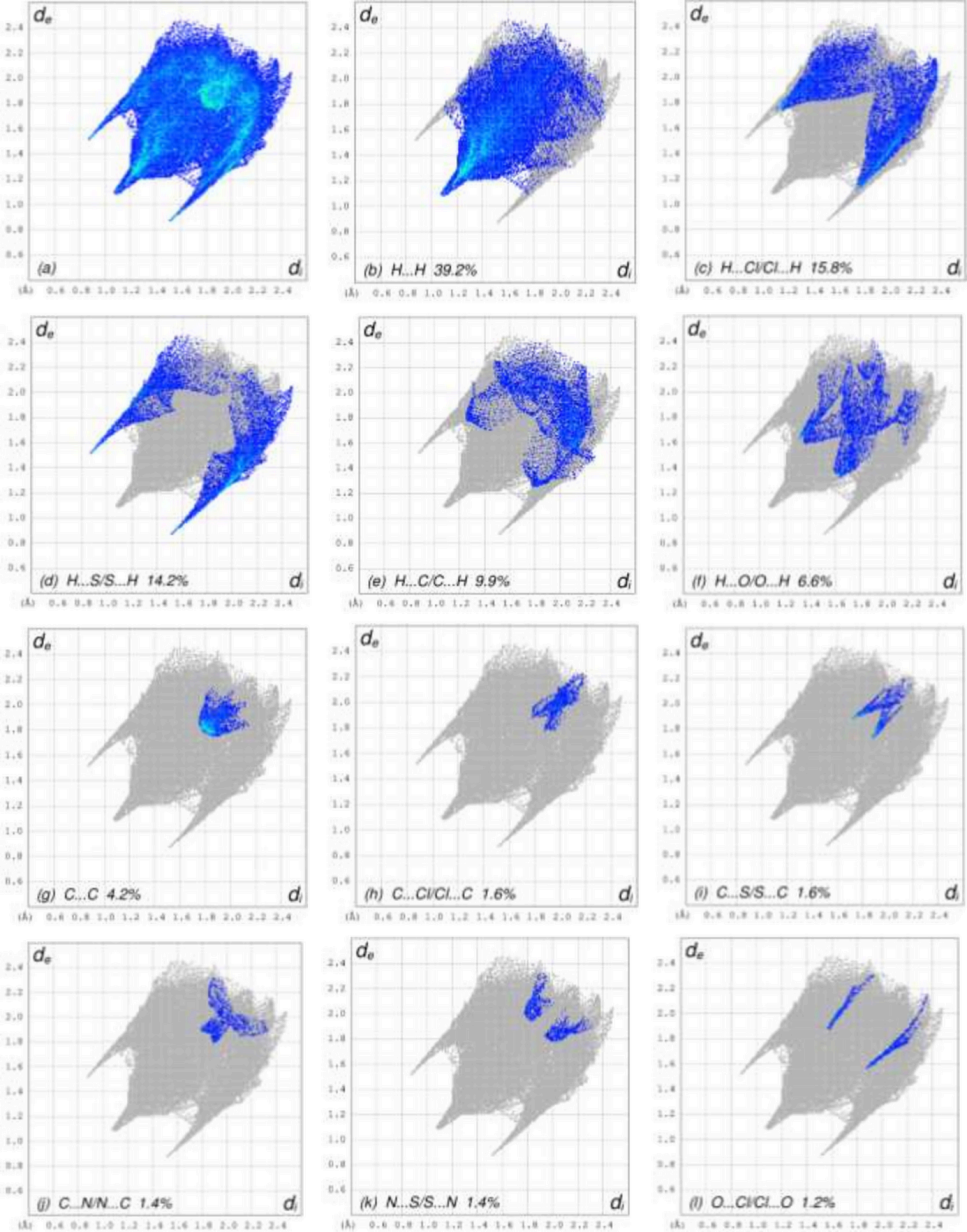
The full two-dimensional fingerprint plots for the title mol­ecule, showing (*a*) all inter­actions, and delineated into (*b*) H⋯H, (*c*) H⋯Cl/Cl⋯H, (*d*) H⋯S/S⋯H, (*e*) H⋯C/C⋯H, (*f*) H⋯O/O⋯H, (*g*) C⋯C, (*h*) C⋯Cl/Cl⋯C, (i) C⋯S/S⋯C, (*j*) C⋯N/N⋯C, (*k*) N⋯S/S⋯N, (*l*) O⋯Cl/Cl⋯O, (*m*) H⋯N/N⋯H, (*n*) C⋯O/O⋯C, (*o*) N⋯O/O⋯N, (*p*) O⋯O and (*r*) S⋯S inter­actions. The *d*_i_ and *d*_e_ values are the closest inter­nal and external distances (in Å) from given points on the Hirshfeld surface.

The overall two-dimensional fingerprint plot is shown in Fig. 5 ▸*a* and those delineated into H⋯H, H⋯Cl/Cl⋯H, H⋯S/S⋯H, H⋯C/C⋯H, H⋯O/O⋯H, C⋯C, C⋯Cl/Cl⋯C, S⋯C/C⋯S, N⋯C/C⋯N, N⋯S/S⋯N, O⋯Cl/Cl⋯O, H⋯N/N⋯H, O⋯O and S⋯S inter­actions are illustrated in Fig. 5 ▸*b*–*r*, respectively. According to the two-dimensional fingerprint plots, the H⋯H, H⋯Cl/Cl⋯H, H⋯S/S⋯H and H⋯C/C⋯H contacts make the most significant contributions to the HS, at 39.2%, 15.8%, 14.2% and 9.9%, respectively (Fig. 5 ▸).

**Figure 6:**
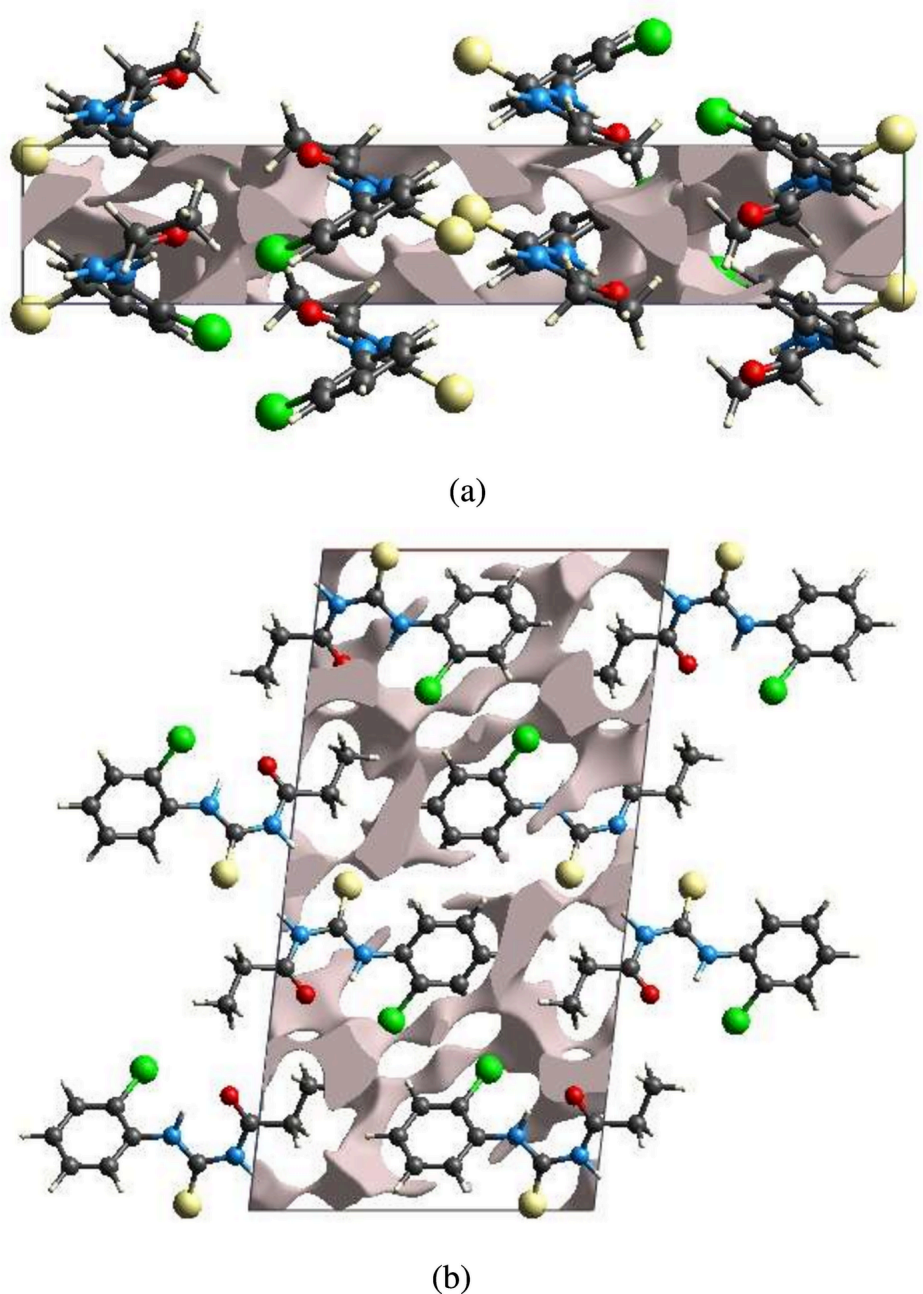
Crystal voids viewed down the (*a*) *a*-axis and (*b*) *b*-axis directions.

The strength of the crystal packing depends on the tight packing of the mol­ecules, which results insignificant voids. To check the strength of the crystal, a void analysis was performed. The volume of the crystal voids (Fig. 6 ▸*a*,*b*) and the percentage of free space in the unit cell were calculated as 105.62 Å^3^ and 9.33%, respectively. Thus, the crystal packing appears compact.

The inter­molecular inter­action energies are calculated using CE–B3LYP/6–31G(d,p) energy model available in *CrystalExplorer17.5* (Spackman *et al.*, 2021 ▸), where a cluster of mol­ecules is generated by applying crystallographic symmetry operations with respect to a selected central mol­ecule within the radius of 3.8 Å by default. The total inter­molecular energy (*E*_tot_) is the sum of electrostatic (*E*_ele_), polarization (*E*_pol_), dispersion (*E*_dis_) and exchange-repulsion (*E*_rep_) energies (Turner *et al.*, 2015 ▸) with scale factors of 1.057, 0.740, 0.871 and 0.618, respectively (Mackenzie *et al.*, 2017 ▸). The hydrogen-bonding inter­action energies (in kJ mol^−1^) for N2—H2*N⋯*S1 were calculated to be −70.5 (*E*_ele_), −12.5 (*E*_pol_), −19.9 (*E*_dis_), 77.0 (*E*_rep_) and −53.5 (*E*_tot_).

**Figure 7:**
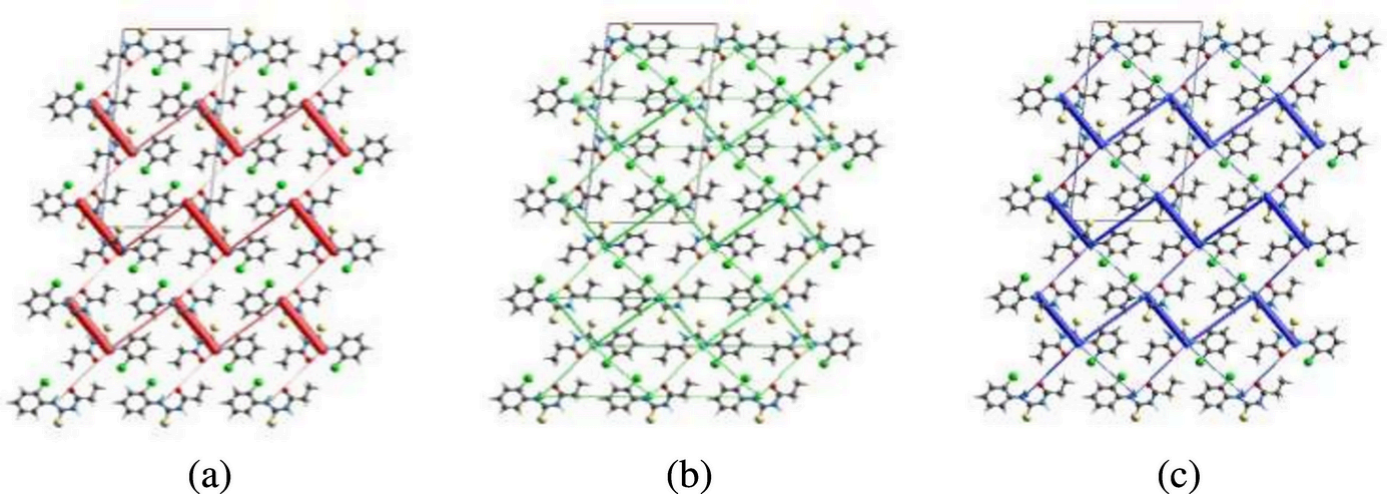
The energy frameworks for a cluster of mol­ecules viewed down the *b*-axis showing the (*a*) electrostatic energy, (*b*) dispersion energy and (*c*) total energy diagrams. The cylindrical radius is proportional to the relative strength of the corresponding energies and they were adjusted to the same scale factor of 80 with cut-off value of 5 kJ mol^−1^ within 2 × 2 × 2 unit cells.

Energy frameworks combine the calculation of inter­molecular inter­action energies with a graphical representation of their magnitudes. They were constructed for *E*_ele_ (red cylinders), *E*_dis_ (green cylinders) and *E*_tot_ (blue cylinders) (Fig. 7 ▸*a*,*b*,*c*). Evaluation of the electrostatic, dispersion and total energy frameworks indicates that the stabilization of the crystal structure is dominated by the electrostatic energy contributions.

## 4. Synthesis and crystallization

*N*-(2-Chloro­phen­yl)-*N*′-propano­ylthio­urea was synthesized by adding dropwise a solution of propinoyl chloride (0.10 mol) in acetone (30 ml) to a suspension of ammonium thio­cyanate (0.10 mol) in acetone (30 ml), and then stirred for 2 h. The reaction mixture was then refluxed for 30 min. After cooling to room temperature, a solution of 2-chloro­aniline (0.10 mol) in acetone (10 ml) was added and the solution refluxed for 3 h. After completion of the reaction (monitored by TLC), the reaction mixture was poured into acidified cold water. The precipitate was filtered under suction, washed with water and dried under vacuum. Colourless crystals suitable for X-ray analysis were obtained by slow evaporation of aceto­nitrile solution. White solid, yield 84%, m.p. 393–394 K. ^1^H NMR (400 MHz, CDCl_3_): δ (ppm) 12.30 (*s*, 1H), 9.77 (*s*, 1H), 7.23–7.61 (*m*, 4H, Ar-H), 1.45 (*t*, 3H, CH_3_), 2.10 (*q*, 2H, CH_2_). ^13^C NMR (100 MHz, CDCl_3_): δ (ppm) 24.4, 31.3, 124.0, 127.0, 128.0, 132.9, 137.4, 141.2, 171.0, 178.7.

## 5. Refinement

Crystal data, data collection and structure refinement details are summarized in Table 2 ▸. The NH hydrogen atoms were located from difference-Fourier maps and refined isotropically. The C-bound H-atom positions were calculated geometrically at distances of 0.93 Å (for aromatic CH), 0.97 Å (for methyl­ene CH) and 0.96 Å (for methyl CH) and refined using a riding model applying the constraint *U*_iso_(H) = *k* × *U*_eq_(C), where *k* = 1.5 for methyl hydrogens and 1.2 for the other H atoms.

## Supplementary Material

Crystal structure: contains datablock(s) I, global. DOI: 10.1107/S205698902600602X/tx2112sup1.cif

Structure factors: contains datablock(s) I. DOI: 10.1107/S205698902600602X/tx2112Isup2.hkl

Supporting information file. DOI: 10.1107/S205698902600602X/tx2112Isup3.cml

CCDC reference: 1816812

Additional supporting information:  crystallographic information; 3D view; checkCIF report

## Figures and Tables

**Table 1:** Hydrogen-bond geometry (Å, °)

*D*—H⋯*A*	*D*—H	H⋯*A*	*D*⋯*A*	*D*—H⋯*A*
N1—H1*N*⋯O1	0.86 (2)	1.92 (2)	2.632 (3)	140 (3)
N2—H2*N*⋯S1^i^	0.85 (2)	2.57 (2)	3.412 (2)	171 (3)

Symmetry code: (i) 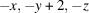.

**Table 2:** Experimental details

Crystal data	
Chemical formula	C_10_H_11_ClN_2_OS
*M*_r_	242.72
Crystal system, space group	Monoclinic, *P*2_1_/*c*
Temperature (K)	293
*a*, *b*, *c* (Å)	11.971 (1), 4.1187 (6), 23.100 (2)
β (°)	96.44 (1)
*V* (Å^3^)	1131.8 (2)
*Z*	4
Radiation type	Mo *K*α
μ (mm^−1^)	0.50
Crystal size (mm)	0.50 × 0.48 × 0.36

Data collection	
Diffractometer	Oxford Diffraction Xcalibur with Sapphire CCD detector
Absorption correction	Multi-scan (*CrysAlis RED*; Oxford Diffraction, 2006 ▸)
*T*_min_, *T*_max_	0.790, 0.842
No. of measured, independent and observed [*I* > 2σ(*I*)] reflections	3971, 2291, 1809
*R*_int_	0.012
(sin θ/λ)_max_ (Å^−1^)	0.625

Refinement	
*R*[*F*^2^ > 2σ(*F*^2^)], *wR*(*F*^2^), *S*	0.043, 0.113, 1.10
No. of reflections	2291
No. of parameters	143
No. of restraints	2
H-atom treatment	H atoms treated by a mixture of independent and constrained refinement
Δρ_max_, Δρ_min_ (e Å^−3^)	0.31, −0.25

Computer programs: *CrysAlis CCD* and *CrysAlis RED* (Oxford Diffraction, 2006 ▸), *SHELXT2014/5* (Sheldrick, 2015*a* ▸), *SHELXL2018/3* (Sheldrick, 2015*b* ▸), *ORTEP-3 for Windows* and *WinGX* publication routines (Farrugia, 2012 ▸) and *PLATON* (Spek, 2020 ▸).

## supplementary crystallographic information

### N-(2-Chlorophenyl)-N'-propanoylthiourea Crystal data

**Table:**

C_10_H_11_ClN_2_OS	*F*(000) = 504
*M**_r_* = 242.72	*D*_x_ = 1.424 Mg m^−^^3^
Monoclinic, *P*2_1_/*c*	Mo *K*α radiation, λ = 0.71073 Å
*a* = 11.971 (1) Å	Cell parameters from 2737 reflections
*b* = 4.1187 (6) Å	θ = 2.6–27.6°
*c* = 23.100 (2) Å	µ = 0.50 mm^−^^1^
β = 96.44 (1)°	*T* = 293 K
*V* = 1131.8 (2) Å^3^	Prism, colorless
*Z* = 4	0.50 × 0.48 × 0.36 mm

### N-(2-Chlorophenyl)-N'-propanoylthiourea Data collection

**Table:**

Oxford Diffraction Xcalibur with Sapphire CCD detector diffractometer	1809 reflections with *I* > 2σ(*I*)
Rotation method data acquisition using ω scans.	*R*_int_ = 0.012
Absorption correction: multi-scan (CrysAlis RED; Oxford Diffraction, 2006)	θ_max_ = 26.4°, θ_min_ = 2.6°
*T*_min_ = 0.790, *T*_max_ = 0.842	*h* = −14→11
3971 measured reflections	*k* = −3→5
2291 independent reflections	*l* = −21→28

### N-(2-Chlorophenyl)-N'-propanoylthiourea Refinement

**Table:**

Refinement on *F*^2^	2 restraints
Least-squares matrix: full	Hydrogen site location: mixed
*R*[*F*^2^ > 2σ(*F*^2^)] = 0.043	H atoms treated by a mixture of independent and constrained refinement
*wR*(*F*^2^) = 0.113	*w* = 1/[σ^2^(*F*_o_^2^) + (0.0471*P*)^2^ + 0.6068*P*] where *P* = (*F*_o_^2^ + 2*F*_c_^2^)/3
*S* = 1.10	(Δ/σ)_max_ < 0.001
2291 reflections	Δρ_max_ = 0.31 e Å^−^^3^
143 parameters	Δρ_min_ = −0.25 e Å^−^^3^

### N-(2-Chlorophenyl)-N'-propanoylthiourea Special details

**Table:**

Geometry. All esds (except the esd in the dihedral angle between two l.s. planes) are estimated using the full covariance matrix. The cell esds are taken into account individually in the estimation of esds in distances, angles and torsion angles; correlations between esds in cell parameters are only used when they are defined by crystal symmetry. An approximate (isotropic) treatment of cell esds is used for estimating esds involving l.s. planes.

### N-(2-Chlorophenyl)-N'-propanoylthiourea Fractional atomic coordinates and isotropic or equivalent isotropic displacement parameters (Å^2^)

**Table:**

	*x*	*y*	*z*	*U*_iso_*/*U*_eq_	
Cl1	0.35101 (6)	1.1838 (2)	0.21334 (3)	0.0615 (2)	
S1	0.18007 (5)	1.06937 (19)	0.00913 (3)	0.0500 (2)	
O1	0.09369 (15)	0.5612 (6)	0.17325 (7)	0.0683 (6)	
N1	0.24754 (16)	0.8040 (6)	0.11267 (8)	0.0434 (5)	
N2	0.05779 (16)	0.7857 (6)	0.08305 (8)	0.0414 (5)	
C1	0.36338 (18)	0.8729 (6)	0.11114 (9)	0.0381 (5)	
C2	0.4218 (2)	1.0438 (6)	0.15665 (9)	0.0402 (6)	
C3	0.5355 (2)	1.1061 (7)	0.15738 (11)	0.0516 (7)	
H3	0.573956	1.219918	0.188205	0.062*	
C4	0.5911 (2)	0.9986 (7)	0.11229 (11)	0.0548 (7)	
H4	0.667268	1.042699	0.112239	0.066*	
C5	0.5347 (2)	0.8257 (7)	0.06703 (11)	0.0540 (7)	
H5	0.572970	0.751932	0.036720	0.065*	
C6	0.4216 (2)	0.7618 (7)	0.06657 (10)	0.0466 (6)	
H6	0.384135	0.643217	0.036089	0.056*	
C7	0.16460 (19)	0.8754 (6)	0.07130 (9)	0.0375 (5)	
C8	0.02653 (19)	0.6352 (7)	0.13210 (10)	0.0435 (6)	
C9	−0.0974 (2)	0.5694 (7)	0.13048 (10)	0.0487 (6)	
H9A	−0.118402	0.402049	0.101768	0.058*	
H9B	−0.138509	0.764965	0.118118	0.058*	
C10	−0.1317 (2)	0.4623 (9)	0.18860 (12)	0.0687 (9)	
H10A	−0.111548	0.627255	0.217244	0.103*	
H10B	−0.093709	0.263737	0.200384	0.103*	
H10C	−0.211450	0.427960	0.185048	0.103*	
H1N	0.227 (3)	0.712 (7)	0.1431 (10)	0.082*	
H2N	0.004 (2)	0.821 (8)	0.0569 (11)	0.082*	

### N-(2-Chlorophenyl)-N'-propanoylthiourea Atomic displacement parameters (Å^2^)

**Table:**

	*U*^11^	*U*^22^	*U*^33^	*U*^12^	*U*^13^	*U*^23^
Cl1	0.0633 (5)	0.0837 (6)	0.0373 (3)	0.0074 (4)	0.0045 (3)	−0.0067 (3)
S1	0.0348 (3)	0.0719 (5)	0.0415 (3)	−0.0044 (3)	−0.0031 (2)	0.0171 (3)
O1	0.0401 (10)	0.1185 (19)	0.0455 (10)	0.0024 (11)	0.0009 (8)	0.0306 (11)
N1	0.0303 (10)	0.0661 (15)	0.0327 (10)	−0.0021 (10)	−0.0020 (8)	0.0078 (10)
N2	0.0293 (10)	0.0591 (13)	0.0348 (10)	0.0000 (10)	−0.0015 (7)	0.0061 (9)
C1	0.0301 (11)	0.0487 (15)	0.0343 (11)	0.0039 (10)	−0.0015 (9)	0.0078 (10)
C2	0.0384 (13)	0.0499 (15)	0.0312 (11)	0.0041 (11)	−0.0015 (9)	0.0059 (10)
C3	0.0421 (14)	0.0605 (18)	0.0487 (14)	−0.0061 (13)	−0.0105 (11)	0.0023 (13)
C4	0.0298 (13)	0.070 (2)	0.0634 (17)	−0.0012 (13)	0.0018 (11)	0.0100 (15)
C5	0.0426 (14)	0.0710 (19)	0.0498 (14)	0.0126 (14)	0.0112 (11)	0.0060 (14)
C6	0.0400 (13)	0.0608 (17)	0.0382 (12)	0.0042 (12)	0.0004 (10)	−0.0028 (12)
C7	0.0340 (12)	0.0453 (14)	0.0322 (11)	0.0009 (10)	−0.0013 (9)	−0.0009 (10)
C8	0.0382 (13)	0.0564 (17)	0.0356 (12)	0.0023 (12)	0.0032 (10)	0.0035 (11)
C9	0.0368 (13)	0.0617 (17)	0.0474 (13)	−0.0064 (13)	0.0039 (10)	0.0025 (13)
C10	0.0550 (17)	0.099 (3)	0.0533 (16)	−0.0196 (18)	0.0133 (13)	0.0054 (17)

### N-(2-Chlorophenyl)-N'-propanoylthiourea Geometric parameters (Å, º)

**Table:**

Cl1—C2	1.736 (2)	C3—H3	0.9300
S1—C7	1.672 (2)	C4—C5	1.378 (4)
O1—C8	1.213 (3)	C4—H4	0.9300
N1—C7	1.331 (3)	C5—C6	1.377 (3)
N1—C1	1.420 (3)	C5—H5	0.9300
N1—H1N	0.858 (17)	C6—H6	0.9300
N2—C8	1.380 (3)	C8—C9	1.504 (3)
N2—C7	1.387 (3)	C9—C10	1.513 (3)
N2—H2N	0.847 (17)	C9—H9A	0.9700
C1—C6	1.384 (3)	C9—H9B	0.9700
C1—C2	1.387 (3)	C10—H10A	0.9600
C2—C3	1.384 (3)	C10—H10B	0.9600
C3—C4	1.371 (4)	C10—H10C	0.9600
			
C7—N1—C1	126.15 (19)	C5—C6—C1	120.5 (2)
C7—N1—H1N	115 (2)	C5—C6—H6	119.8
C1—N1—H1N	118 (2)	C1—C6—H6	119.8
C8—N2—C7	128.42 (19)	N1—C7—N2	115.49 (19)
C8—N2—H2N	114 (2)	N1—C7—S1	125.21 (18)
C7—N2—H2N	118 (2)	N2—C7—S1	119.27 (16)
C6—C1—C2	118.6 (2)	O1—C8—N2	122.6 (2)
C6—C1—N1	121.8 (2)	O1—C8—C9	122.6 (2)
C2—C1—N1	119.6 (2)	N2—C8—C9	114.7 (2)
C3—C2—C1	121.0 (2)	C8—C9—C10	113.3 (2)
C3—C2—Cl1	119.47 (19)	C8—C9—H9A	108.9
C1—C2—Cl1	119.52 (18)	C10—C9—H9A	108.9
C4—C3—C2	119.4 (2)	C8—C9—H9B	108.9
C4—C3—H3	120.3	C10—C9—H9B	108.9
C2—C3—H3	120.3	H9A—C9—H9B	107.7
C3—C4—C5	120.3 (2)	C9—C10—H10A	109.5
C3—C4—H4	119.8	C9—C10—H10B	109.5
C5—C4—H4	119.8	H10A—C10—H10B	109.5
C6—C5—C4	120.1 (2)	C9—C10—H10C	109.5
C6—C5—H5	119.9	H10A—C10—H10C	109.5
C4—C5—H5	119.9	H10B—C10—H10C	109.5
			
C7—N1—C1—C6	−56.9 (4)	C2—C1—C6—C5	−1.2 (4)
C7—N1—C1—C2	126.0 (3)	N1—C1—C6—C5	−178.4 (2)
C6—C1—C2—C3	0.8 (4)	C1—N1—C7—N2	−179.4 (2)
N1—C1—C2—C3	178.0 (2)	C1—N1—C7—S1	−1.3 (4)
C6—C1—C2—Cl1	−179.63 (19)	C8—N2—C7—N1	0.6 (4)
N1—C1—C2—Cl1	−2.4 (3)	C8—N2—C7—S1	−177.7 (2)
C1—C2—C3—C4	0.3 (4)	C7—N2—C8—O1	0.4 (5)
Cl1—C2—C3—C4	−179.3 (2)	C7—N2—C8—C9	−179.5 (2)
C2—C3—C4—C5	−1.0 (4)	O1—C8—C9—C10	10.7 (4)
C3—C4—C5—C6	0.5 (4)	N2—C8—C9—C10	−169.4 (3)
C4—C5—C6—C1	0.6 (4)		

### N-(2-Chlorophenyl)-N'-propanoylthiourea Hydrogen-bond geometry (Å, º)

**Table:**

*D*—H···*A*	*D*—H	H···*A*	*D*···*A*	*D*—H···*A*
N1—H1*N*···O1	0.86 (2)	1.92 (2)	2.632 (3)	140 (3)
N2—H2*N*···S1^i^	0.85 (2)	2.57 (2)	3.412 (2)	171 (3)

Symmetry code: (i) −*x*, −*y*+2, −*z*.
